# Design and Control of Dual-Segment Multi-Wire Driven Bionic Soft Arm with Integrated Suction Cups

**DOI:** 10.3390/biomimetics10030133

**Published:** 2025-02-24

**Authors:** Zhaosheng Wu, Qiuxuan Wu, Fulin Du, Zikai Zhao, Shoucheng Xiang, Hongkun Zhou, Yanbin Luo, Zhiyuan Hu

**Affiliations:** 1 HDU-ITMO Joint Institute, Hangzhou Dianzi University, Hangzhou 310018, China; 2 School of Automation, Hangzhou Dianzi University, Hangzhou 310018, China; 3 International Joint Research Laboratory for Autonomous Robotic Systems, Hangzhou Dianzi University, Hangzhou 310018, China; 4 Kunming Shipborne Equipment Research and Test Center, Kunming 650106, China

**Keywords:** underwater soft arm, soft robot, wire drive, kinematic modeling, bionic suction cups

## Abstract

Given the growing complexity of underwater operation tasks, particularly in confined spaces, turbulent environments, and dynamic object manipulation, the limitations of traditional rigid robotic arms are becoming ever more evident. To tackle these challenges, this paper proposes the development of a soft robotic arm modeled after octopus tentacles, incorporating biomimetic suckers. To tackle these challenges, this paper proposes the development of a soft robotic arm modeled after octopus tentacles, incorporating biomimetic suckers. By imitating the functional structure and suction cups of an octopus arm, a soft arm with a dual-segment continuous structure and eight-wire drive control is designed, integrating a flexible suction cup at the distal segment. A three-dimensional, dual-segment eight-wire driven segmented constant curvature motion model is developed to enable precise bending and rotational movements. In underwater grasping experiments, the soft robotic arm exhibited enhanced grasping stability, particularly in underwater environments, where it effectively copes with fluid disturbances and the capture of dynamic objects. This substantially increased the reliability and efficiency of underwater operations.

## 1. Introduction

Soft robots, due to their high flexibility, high compliance, and strong interaction ability with unstructured environments, have created new possibilities for task execution in complex settings [1,2]. Particularly in the field of underwater operations, complex environments (such as narrow gaps, turbulent water flow, and irregular surfaces) require robots to have high flexibility and adaptability [3]. In marine exploration, underwater archaeology, and rescue missions [4], traditional rigid robotic arms, due to their high structural rigidity and limited adaptability to complex environments, show significant limitations in underwater grasping and handling tasks [5,6]. The octopus arm [7,8], as a natural model, provides significant inspiration for the design of underwater soft robots due to its excellent multi-degree-of-freedom motion and adhesion capabilities. Although previous studies have developed efficient underwater grasping robots by mimicking the negative pressure adhesion principle of octopus suckers [9], current technologies still face challenges in achieving flexible manipulation and stable adhesion in complex underwater environments [10]. Biomimetic soft arms continue to face several challenges in both structural design and performance enhancement. For example, the grasping force and stability of the flexible structure are insufficient in high-load tasks; and, the dynamic response performance of the suction cup adhesion function still requires optimization in complex underwater environments. This presents new research demands for the further development of bio-inspired soft arms integrating both flexible structures and adhesion functions. Some flexible robotic arms are constructed using purely flexible materials (e.g., silicone or elastomers) [11], which provides outstanding compliance and environmental adaptability, but their performance is often constrained by the insufficient material strength in high-load tasks. For instance, the silicone-based flexible robotic arm driven by a shape memory alloy (SMA) coil, as designed by Haoyang et al. [12], experiences noticeable deformation due to stress concentration during high-load tasks, resulting in task failure. The cable-driven bio-inspired continuum robot designed by Azamat Yeshmukhametov [13], uses a multi-joint rigid skeletal structure, which enhances load-bearing capacity compared to purely flexible robotic arms [14]; however, this design shows limited performance in adapting to complex terrains and achieving non-damaging contact. Therefore, neither purely flexible nor rigid designs can fully meet the demands of complex tasks [15]. Rigid-flexible coupling technology has gradually become an important trend in addressing the insufficient load capacity of soft robots.

The driving technology of soft robotic arms plays a key role in improving their performance and versatility. Currently, the main driving methods include cable-driven [16], fluid-driven, and smart material-driven systems. Cable-driven technology uses internal cables to pull flexible materials, providing advantages like a simple structure and ease of achieving multi-degree-of-freedom movement. However, it faces the problem of cable slack. Fluid actuation technology (pneumatic and hydraulic) is suitable for high-load tasks, but its large size and high sealing requirements pose limitations in miniaturization and portability. Smart material-driven technologies (such as shape memory alloys and dielectric elastomers) offer advantages in miniaturization and responsiveness, but they have limited driving force and durability issues. To overcome the limitations of single actuation technologies, researchers have gradually turned their attention to hybrid actuation technologies [17]. Yara Almubarak et al. proposed a new type of underwater robot, Kraken [18], which employs a hybrid drive technology made up of a stepper motor and twisted polymer fishing line muscles (TCPFL), enabling various movement modes, including curling, twisting, and bending. This design showcases the immense potential of hybrid drive technology in improving the performance of soft robotic arms.

In recent years, biomimetic suction cups have been extensively used in the design of flexible robotic arms. The octopus suction cup, with its unique coxo-pectoral structure [19], resembling a muscle-hydraulic system, has been widely mimicked. Frey et al. developed a controllable octopus suction cup with freely adjustable surface adhesion [20]. It can securely attach to various surfaces while achieving quick suction and release. Zhexin Xie et al. [21] integrated biomimetic suction cups into a flexible robotic arm and developed a suction cup-equipped biomimetic octopus arm. By optimizing the cone angle design, they improved both the grasping force and stability of the arm. Biomimetic designs, inspired by natural organisms such as octopuses, have greatly enhanced their adhesion mechanisms. Li et al. [22] proposed a composite biomimetic adhesive surface, which combines features of wedge-shaped, sucker, and spine-like surfaces, demonstrating superior adaptability to various surface roughness and curvatures. Wu et al. [23] proposed a bioinspired suction cup with adaptive grasping capabilities, suitable for capturing irregular objects in complex underwater environments. These findings highlight the significant role of bioinspired suction cups in high-dynamic environments.

This paper designs a novel soft arm based on suction cup adhesion, featuring a two-segment serial continuous structure. It is primarily actuated by cables, with eight drive cables controlled by four waterproof servos. The soft arm is made by casting silicone into a 3D-printed mold. Kinematic analysis is performed using a piecewise constant curvature model, enabling precise control of the motion trajectory.

## 2. Design and Fabrication

### 2.1. Bio-Inspiration

**Figure 1:**
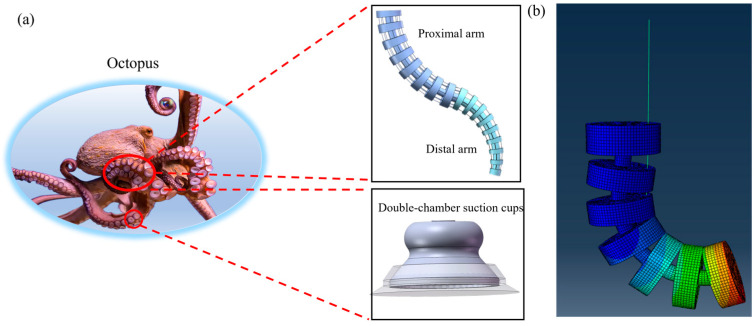
(**a**) Inspiration from octopus in nature, (**b**) Stress contour map of the structural simulation.

Octopuses are capable of flexible grasping, movement, and manipulation (see Figure 1). Inspired by this, a soft robotic arm integrated with suction cups is designed.

### 2.2. Design and Simulation of the Soft Robotic Arm

**Figure 2:**
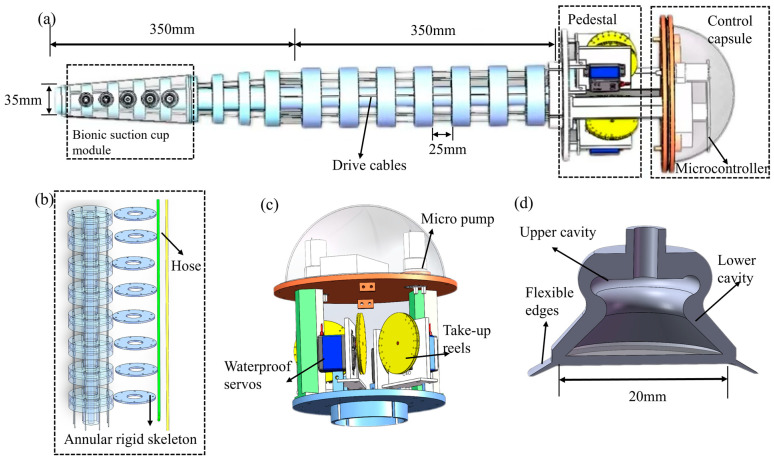
Soft robotic arm prototype integrated with biomimetic suction cups. (**a**) Overall prototype structure; (**b**) Composition of the proximal arm; (**c**) Control and drive system; (**d**) Biomimetic octopus suction cup.

A two-segment serial soft robotic arm, combined with biomimetic suction cups (see Figure 2a), was designed to address the operational grasping requirements in complex underwater environments. First, structural simulation analysis was conducted using ABAQUS(2022) software, including the relevant characteristics of the soft arm, such as bending deformation, end displacement, and end forces. The analysis focused on bending deformation and associated strain and stress to replicate the complex movement of the octopus arm. The simulation uses a single cable to apply a smooth concentrated force of 10 N, triggering the bending of the soft arm. (See Figure 1b the cloud map indicates that the soft arm can achieve a bending angle of around 90 degrees. The deformation contour plot illustrates the stress distribution the soft arm undergoes during bending, and the results show that the structure meets the design specifications.

The overall structure of the soft arm consists of two segments: the proximal and distal segments, with a modular design, where each segment is independently driven and controlled. The proximal arm (length: 350 mm, diameter: 80 mm) is made up of several segments of continuous silicone, spaced 25 cm apart. An annular rigid skeleton is embedded inside to provide the required support, and the arm is molded with highly flexible silicone material. It is worth noting that the hollow design of the soft arm is intended to accommodate hydraulic pipelines, providing an efficient fluid transmission path for the suction cup system (see Figure 2b). The distal arm segment (length: 350 mm, end radius: 15 mm) is designed as a conical continuum structure, replicating the bending flexibility of the octopus arm. The integration of the biomimetic octopus suction cup with the distal arm is accomplished through a flexible mounting structure, enabling effective coordination.

The drive system of the soft arm utilizes cable-driven actuation, with eight evenly spaced through holes (inner diameter: 2 mm) embedded in the proximal arm, allowing the soft arm to perform multidirectional bending movements in space. The through holes are divided into two groups: four of them pass through the drive cables of the first segment, and the remaining four are used for the drive cables of the second segment. The cables are connected to the winding drums on the waterproof servos (PDI-6221MG, from Shantou Extreme Technology Co., Ltd., Shantou, China). The spacing between adjacent drive cables is 45°, while the spacing between two cables in the same segment is 90°. When the waterproof servo drives the winding drum to rotate, it changes the length of the drive cables, thereby controlling the soft arm to bend in different directions and allowing the soft arm to bend flexibly in three-dimensional space. Mounted on the base platform, it is used to secure the four servos and the soft arm.

A dual-chamber biomimetic suction cup is installed on the outer side of the distal arm (see Figure 2d), with the overall structure made up of an inner chamber and an outer chamber. The outer chamber is responsible for initial contact with the target surface and forming a seal, while the inner chamber enhances adhesion by adjusting the pressure differential. The suction cups are designed and manufactured by mimicking the geometric shapes of the octopus’s funnel and coxo-pectoral structures, with suction cups having diameters of 20 mm and 16 mm, respectively. Each suction cup generates a suction force of 5 N and is fixed with 5 mm diameter slots. Five suction cups are arranged vertically. The suction cup is made from flexible materials, comprising an upper chamber, a lower chamber, and a flexible edge. The flexible edge design enables adaptation to irregular surfaces, ensuring a strong sealing effect. It enables vacuum suction and release functionality. The control system is integrated into the base (see Figure 2c), sealed with a hemispherical acrylic plate and O-ring, and houses the central control module, power module, and micro water pump drive unit. The base is securely connected to the proximal arm via a circular bayonet, with drive signals and power transmitted through cables to the arm segments and suction cup units.

### 2.3. Fabrication of the Soft Arm

**Figure 3:**
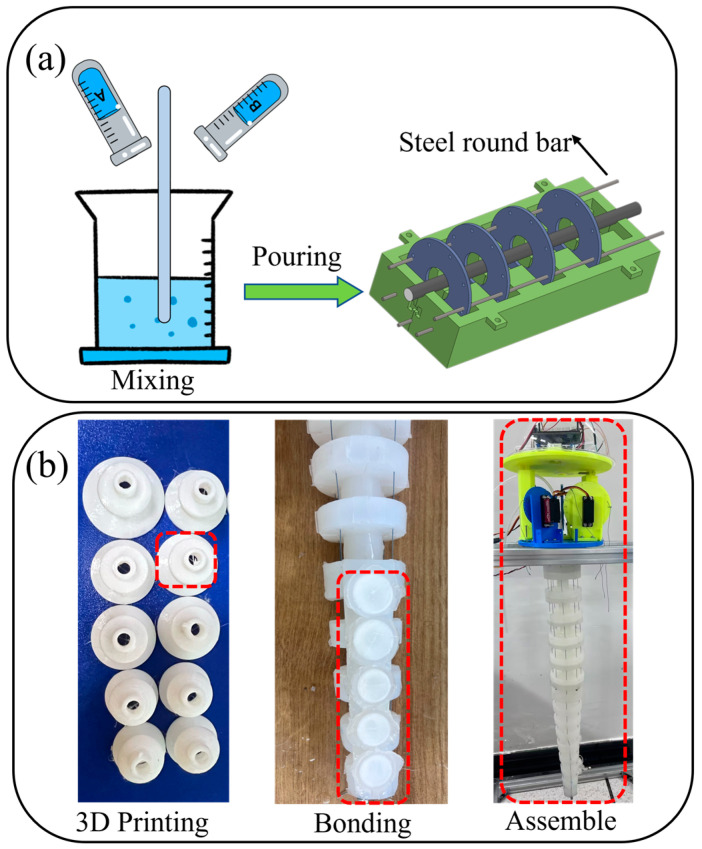
Soft arm prototype fabrication process. (**a**) Soft arm fabrication flowchart, (**b**) Biomimetic octopus suction cup fabrication flowchart.

The molds and rigid parts of the biomimetic octopus arm were designed in SolidWorks and produced using 3D printing technology. The 3D printing was performed using the CR-10 Smart Pro printer, provided by Creality 3D, located in Shenzhen, China. Due to the difficulty of casting the entire structure in one piece, the mold for each segment of the soft arm is divided into four parts, and casting is performed in stages. First, the annular rigid skeleton is fixed at equal intervals into the proximal arm mold, then a 3 mm diameter high-speed steel rod is embedded into the circular holes reserved on the skeleton (see Figure 3a). Due to the varying dimensions of each segment, a custom mold must be made for each one. The silicone used is the POSILICONE brand A/B series, manufactured by Puston Materials Co., located in Shenzhen, China, which has self-degassing properties. The silicone has the following properties: Shore hardness of 40 A°, density of 1.1 g/cm^3^, viscosity of 2000 Pa s, and curing time of 4–5 h. After the A/B silicone mixture is fully stirred, it is placed in a vacuum chamber to generate a negative pressure environment, preventing bubble entrapment. The mixture is cast into the mold, and, after curing, the steel rod is extracted and the edges are trimmed. The drive cables are made of high-strength Kevlar wire, fixed at the end of each soft arm segment with a mounting pad. They pass through internal guide channels within the arm and are secured to the servo winding drum in the base. The biomimetic suction cup is 3D printed directly from thermoplastic TPU elastomer, and then the flexible edge is permanently bonded to the suction cup to achieve a sealing effect (Figure 3b). The entire suction cup module is constructed by fixing the separately made suction cup and flexible connector into the slots. It is connected to the micro water pump through a hose, which runs through the soft arm’s hollow channels. The side of the distal arm integrates a biomimetic suction cup, with the suction force controlled by a hydraulic system, ensuring excellent grasping ability in underwater environments. The suction cup is made from highly flexible silicone or elastomer materials to ensure its adaptability and durability in underwater environments. The suction cup is driven by a micro water pump within the base, allowing real-time adjustment of the suction force based on operational needs. When the suction cup makes contact with the target surface, the water pump quickly extracts water from inside the suction cup, creating a negative pressure and enabling fast and secure adhesion. This design ensures that the suction cup can effectively meet the gripping needs of different surfaces in diverse and complex underwater environments.

## 3. Kinematic Analysis and Simulation

### 3.1. Kinematic Analysis

**Figure 4:**
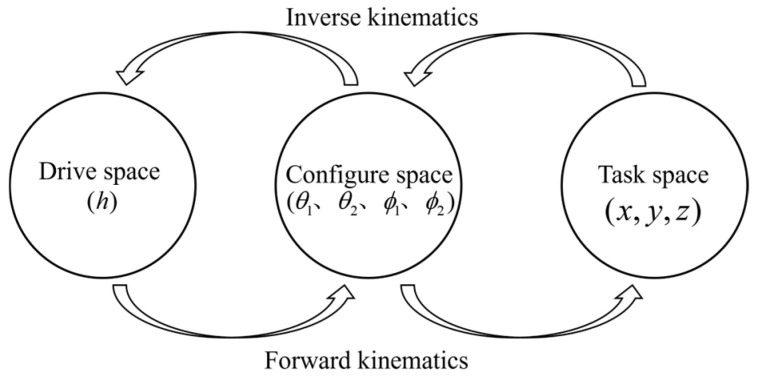
Space Description Diagram.

In this study, a piecewise constant curvature model is employed for the kinematic analysis of the soft arm. It is assumed that the soft arm forms a standard arc during bending, with the bending angle and related parameters remaining constant within each small segment. By establishing the mapping relationship between the drive space, configuration space, and task space, precise control and motion analysis of the soft arm are achieved (See Figure 4).

#### 3.1.1. Kinematic Mapping from Drive Space to Virtual Joint Space

**Figure 5:**
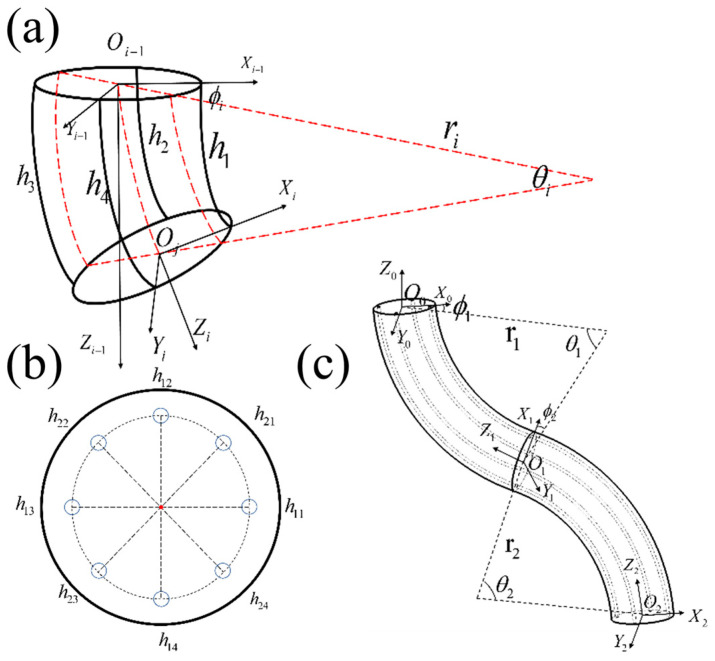
(**a**) Simplified side view of the model, (**b**) Distribution diagram of drive cables, (**c**) Geometric model of the soft arm.

The coordinate system is assigned to the base end of the soft arm. The four-channel flexible actuators directly control the length of the drive cables inside the soft arm $h_{j}=(j=1,2,3,4)$. $\theta_{i}$ represents the curvature angle of the arc, and $\phi_{i}$ represents the bending plane angle. Combined with the side view of the flexible actuator (see Figure 5a).

Since the distal arm is conical, let the radius of the soft arm’s end cross-section be $R_{\min}$ and the radius of the top cross-section be $R_{\max}$. When the soft arm is uniformly divided into n segments, the approximate radius of the top cross-section of the ith segment is

(1) $$R_{i}=R_{\max}-\frac{i}{n}(R_{\max}-R_{\min})$$

The soft arm with a dual-segment continuous structure consists of eight drive cables evenly spaced at 45° intervals (as shown in Figure 5b), with four drive cables grouped together. When the soft arm undergoes both rotational and bending movements, the change in the length of the drive cables can be calculated using the abc formula:

(2) $$\Delta h_{i,j}=(r_{i}-r_{i,j})\theta_{i}=\theta_{i}R_{i}\cos\left(\phi_{i}-(j-1)\frac{\pi }{2}+(i-1)\frac{\pi }{4}\right)$$

$\Delta h_{i,j}$: The length variation in the *j*-th cable in the *i*-th segment
$\theta_{i}$: The bending angle of the *i*-th segment (independent of j)
$r_{i}$: The curvature radius of the circular arc corresponding to segment *i*, $\theta_{i}$
$r_{i,j}$: The curvature radius of the *j*-th cable in the i-th segment
$\phi_{i}$: The bending plane angle of the *i*-th segment
$R_{i}$: The approximate radius of the top cross-section of the *i*-th segment

In the design of the soft arm, linear drive elements are embedded within the internal channels of the arm segments to enable its movement. The drive cables of the distal arm in the soft arm pass through the proximal arm. This design implies that when the proximal arm bends or rotates, it will cause coupling effects on the distal arm through the drive cables. To keep the distal arm stationary, the latter must adjust its drive cables to compensate for the coupling effects caused by the motion of the former. On the other hand, the motion of the distal segment does not influence the drive cables of the proximal segment, indicating that the coupling effect is one-way. The end of the soft arm must achieve multi-dimensional guidance, and its motion needs to be decoupled for analysis. According to Formula (2), the elongation variation formula for each decoupled drive cable can be derived. Substituting these into the calculation allows for the mapping relationship between the drive space and the virtual joint space:

(3) $$\phi_{1}=\arctan\left(\frac{\Delta h_{12}}{\Delta h_{11}}\right)$$

(4) $$\theta_{1}=\frac{\Delta h_{11}}{R_{1}\cos(\phi_{1})}$$

(5) $$\phi_{2}=\arctan\left(\frac{\left(q_{6}-\Delta h_{16})-(q_{5}-\Delta h_{15}\right)}{\left(q_{6}-\Delta h_{16})+(q_{5}-\Delta h_{15}\right)}\right)$$

(6) $$\theta_{2}=\frac{q_{6}-\Delta_{16}}{R_{2}\cos(\phi_{2}+\frac{\pi }{4})}$$

#### 3.1.2. Kinematic Mapping from Virtual Joint Space to Task Space

Based on the segmented constant curvature assumption, once the configuration space parameters are known, the pose problem of the soft arm can be simplified by solving the homogeneous transformation matrix between the adjacent arcs. The relationship between the D-H parameters and the arc parameters is shown in Table 1.

Based on the D-H table, the general link transformation formula for the coordinate system a relative to b can be derived. The homogeneous transformation matrix between the end-effector coordinate system and its root coordinate system for each segment of the soft arm is as follows (where c represents cos, and s represents sin):

(7) Tii−1=Rot(z,φi)Roty,θi2Trans(zi−1,0,0)Roty,−θi2Rot(z,−φi)c2ϕi(cθi−1)+1sϕicϕi(cθi−1)cϕisθiricϕi(1−cθi)sϕicϕi(cθi−1)s2ϕi(cθi−1)+1sϕisθirisϕi(1−cθi)−cϕisθi−sϕisθicθirisθi0001

In this study, a two-segment flexible drive arm was fabricated. The homogeneous transformation matrix of the soft arm can be obtained using the chain rule, and its expression is as follows:

(8) T20=0T1⋅1T2=R0P1=nxoxaxpxnyoyaypynzozazpz0001

The spatial mapping relationship from task space to configuration space is similar to the inverse kinematics solution of traditional serial robots, which involves solving a system of nonlinear equations, making it difficult to obtain an analytical solution.

### 3.2. Kinematic Simulation

**Figure 6:**
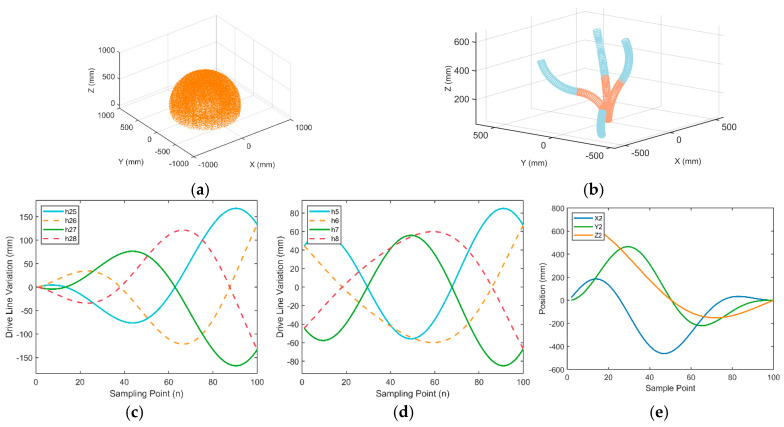
(**a**) Working space of the soft arm, (**b**) Bending motion simulation, (**c**) Changes in the distal drive line when the proximal segment moves independently, (**d**) Distal arm drive line length variation curve, (**e**) Soft arm spatial position diagram.

The operational space of the soft arm reflects the extent of its reachable range. Using the obtained homogeneous transformation matrix T, the coordinate expression of the end-effector reference point can be derived. This study uses the Monte Carlo method to randomly sample points within the bending and rotation range of the soft arm. By plotting the position of each end-point, the working space is obtained (see Figure 6a), indicating that the working space of the soft arm approximates a symmetric spherical canopy space. 

A kinematic simulation was conducted to verify the correctness of the kinematic algorithm. First, four sets of drive cable data were set up in Matlab (2022a) to simulate the bending motion of the soft arm (as shown in Figure 6b). It was found that the soft arm can achieve flexible bending of the arm segment by controlling the elongation variation in the drive cables, verifying the precise control of the target position and orientation by adjusting the drive parameters.

Then, to verify the correctness of the motion mapping between the drive space and configuration space, the relationship between the elongation variation in the drive cables and the configuration space parameter variables was simulated. Let the bending angle be $\left[0,\pi \right]$, the bending plane angle be $\left[0,2\pi \right]$, with 100 sampling points. The soft arm connects the transmission between the two segments, creating a coupling relationship. The coupling relationship between the two segments was analyzed in detail earlier. To better understand the motion process of the soft arm and the variation trends of the drive cables during motion, a simulation was performed. When the first segment moves independently, the length of the transmission system controlling the second segment will change as the joint variables of the first segment vary (as shown in Figure 6c). When the soft arm moves, the motion of the distal arm is affected by the coupling of the proximal arm’s motion, and the motion of the distal arm is the linear superposition of the movements of each segment. Let the bending angle of the proximal arm be $\left[0,\pi \right]$, the bending plane angle be $\left[0,2\pi \right]$, the bending angle of the distal arm be $\left[\pi ,0\right]$, and the bending plane angle be $\left[0,2\pi \right]$. The simulation results are shown in Figure 6d. The motion of the distal segment is significantly influenced by the coupling effect of the motion of the proximal segment, indicating that the coupling effect needs to be fully considered during the design and control of the two segments of the soft arm.

Finally, a simulation analysis of the variation in the end-effector position of the soft arm was performed. The position variation curve of the end-effector reference point during the bending and rotation motion process (see Figure 6e). As seen above, whether it is single-segment motion or coupled motion of the two segments, the simulation results are consistent with the theoretical analysis, indicating that the model can effectively describe the motion behavior of the soft arm.

## 4. Experiment

This study adopts a kinematic algorithm-based control method, Implemented through the STM32F103ZET6 microcontroller development board (produced by STMicroelectronics, with equipment sourced from Geneva, France) to precisely control the movement of the soft robotic arm. The kinematic algorithm is primarily used to compute the joint angles, positions, and velocities of the arm, and to control these kinematic parameters to follow the desired trajectory. The algorithm processes real-time sensor feedback to calculate the necessary control signals and transmits them to the drive system to actuate the arm’s movement. In order to validate the effectiveness of the soft arm mechanics and kinematic analysis methods discussed earlier, as well as its practical capabilities, two prototype arms were created, and the motion performance of the soft arm with a suction cup was tested. The experimental platform consisted of a testing water tank, custom brackets made from aluminum profiles, and a host computer.

### 4.1. Performance Testing

**Figure 7:**
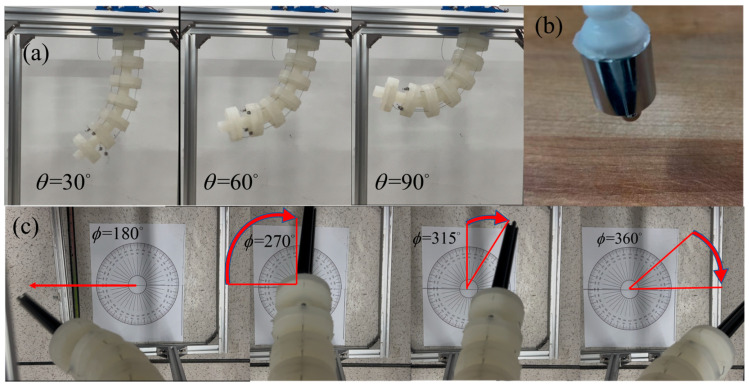
(**a**) Bending experiment diagram under different curvature angles, (**b**) Suction cup lifting a 100 g object, (**c**) Semi-circle trajectory experiment diagram.

**Figure 8:**
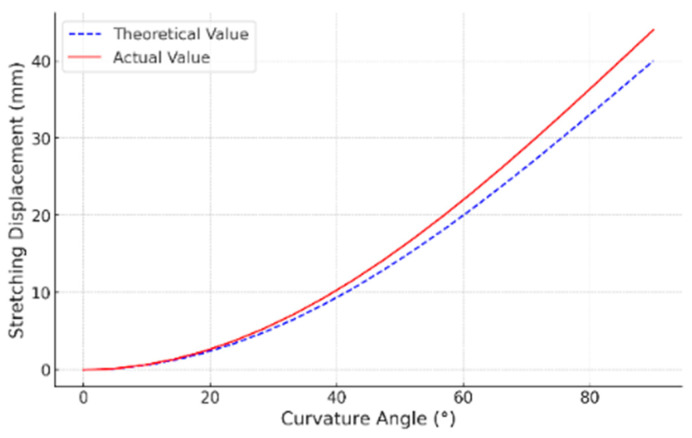
Drive line variation curve.

The first experiment conducted was the single-segment bending performance test. First, a single-segment bending performance test was conducted. By controlling the servo angle, the winding spool was rotated to stretch the drive cables, which in turn caused the soft arm to bend (see Figure 7a). The bionic suction cup was tested afterward and could easily lift a 100 g weight, which improved the stability of the soft arm’s grasp. Finally, by measuring the bending angles at different stretch values and comparing them with the expected theoretical values (see Figure 8), with radius R = 40 mm, bending angle $\theta =\left[0{}^{\circ},90{}^{\circ}\right]$, and bending plane angle ϕ=180°, the experimental results showed that the actual bending angle of the arm was slightly lower than the theoretical value. This discrepancy is due to the fact that the effect of the soft arm’s weight on its bending capacity was not accounted for in the actual tests. In the underwater environment, the buoyancy affects the soft arm, making the bending angle approach the theoretical value. The experimental results validate the accuracy of the earlier simulation analysis, showing that the designed soft arm can achieve the expected motion flexibly.

To further verify the applicability of the kinematic model, the servo rotation angle was adjusted by controlling the configuration space parameters, achieving a semi-circular trajectory motion in the horizontal direction. Set the initial bending plane angle ϕ=180°, bending angle θ=30°, and execute a semi-circular periodic motion of the arm, returning to the initial position when the bending plane angle reaches ϕ=360° (as shown in Figure 7c).

The curvature change during the experimental motion process is consistent with the theoretical expectation. This suggests that the control method obtained through kinematic analysis demonstrates high precision and operability, effectively controlling the motion trajectory of the soft arm. The semi-circular trajectory motion experiment aims to validate the effectiveness of the kinematic model by controlling the configuration space parameters to compute the servo rotation angles, with control applied to one segment. The position information of the marker paper indicates that the designed soft arm can realize the expected bending and rotational motion, thus verifying the validity of the kinematic analysis method and the feasibility of the control method. To verify the kinematic model proposed in this study, we compared it with existing soft robotic and traditional rigid robot models. Although current soft robot models excel in flexibility and adaptability, they exhibit poor stability in high-load and complex tasks.

### 4.2. Underwater S-Shaped Bending Motion Experiment

**Figure 9:**
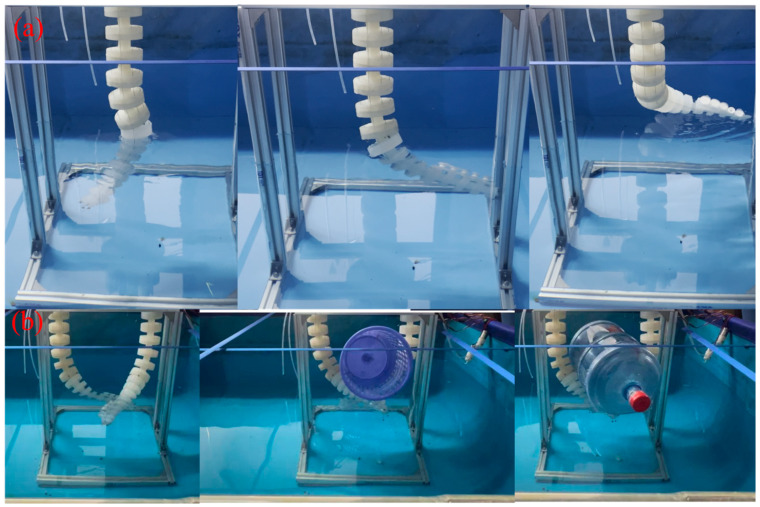
(**a**) Underwater S-shaped bending performance experiment diagram, (**b**) Dual-arm grasping experiment diagram.

To evaluate the flexibility of the soft arm, a single-arm S-shaped bending experiment was conducted in a pool, where the soft arm was able to perform an S-shaped bending motion underwater, and the control parameters were adjusted using a kinematic model to ensure precise motion control (see Figure 9a). Although there was some motion deviation in the experiment, this was primarily due to the coupling effects of multiple factors, including the stiffness of the soft arm, material properties, manufacturing precision, and the pretension force of the drive line. Due to the complexity of interactions among multiple factors, a quantitative analysis is not yet feasible, and therefore, the specific effects of each factor on motion accuracy cannot be precisely quantified. Thus, despite some error, the overall motion performance aligns with the expectation, verifying the high adaptability of the soft arm design.

The experimental results indicate that the accuracy of the soft arm’s motion has some deviation, which is because the designed prototype is a multi-factor coupled system, and its behavior is affected by several factors. Factors such as the overall stiffness, material properties, manufacturing precision, and the pretension of the drive line prevent the soft arm from maintaining a constant curvature during its motion.

### 4.3. Dual-Arm Underwater Grasping Experiment

In the underwater dual-arm embrace grasping experiment, the suction cup adhesion design was incorporated to improve the stability of the object during the grasping process. In the experiment, the soft arm mimicked the embrace motion of an octopus arm through two coupled movements. The suction cup’s adhesion function significantly improved the stability of the object being grasped, especially in the underwater environment, where the suction cup was able to maintain a firm adhesive force, reducing displacement of the grasped object caused by fluid disturbance or motion. The experimental results indicate that the soft arm can accurately complete grasping tasks in a complex underwater environment, and the suction cup’s adhesion greatly enhanced the success rate and stability of the grasping.

## 5. Discussion and Conclusions

This paper introduces the development of a biomimetic octopus soft arm, which successfully performs grasping tasks in complex environments. In comparison with fully soft robotic arms and purely rigid mechanisms, the rigid-skeleton silicon-based soft arm provides high load-bearing capacity while offering good flexibility, making it suitable for complex environments. Fully soft robotic arms excel in adaptability and flexibility but have weak load capacity; purely rigid mechanisms have strong load-bearing capabilities but lack flexibility, which limits their ability to handle challenges in complex environments. The rigid-soft hybrid structure proposed in this paper strikes a good balance between load capacity, flexibility, and control precision, exhibiting outstanding overall performance. Through kinematic analysis and experimental validation, the feasibility of motion control and the adhesion capability of the biomimetic suction cup were verified. This study experimentally confirmed the accuracy of the kinematic model, particularly in bending, semi-circular trajectory, and S-shaped bending motions. The soft arm achieved precise control, and the actual movement aligned closely with the theoretical model. The dual-arm embrace grasping experiment demonstrates that the soft arm can execute complex movements with multiple degrees of freedom and successfully complete efficient grasping tasks.

During the study, we adopted an innovative dual-chamber suction cup design that effectively addresses water flow disturbances and changes in dynamic objects during grasping. In comparison with previous designs, the soft robotic arm developed in this study not only improves grasping stability but also broadens its potential for application in dynamic environments. The advantage of the suction cup design is its adaptability to different surfaces, especially in underwater environments, where it can securely attach to target objects and prevent displacement due to water flow or object movement. The soft robotic arm offers a maximum reach of 70 cm and a maximum payload capacity of 2 kg, enabling stable grasping and handling of most common objects. The arm’s operational speed has been validated in different experimental tasks, meeting the needs of most underwater operations. In terms of structure and materials, the design that combines silicone with a rigid skeleton not only provides the soft arm with high load capacity but also ensures its good compliance and safety in complex underwater environments.

The soft robotic arm design presented in this paper is more flexible and adaptable than traditional rigid robotic arms. In this study, the suction cup design was optimized to allow the soft robotic arm to better adapt to complex environments, particularly under conditions of high load or significant fluid disturbances. However, this study has some limitations. For instance, although the soft robotic arm performs well under the current experimental conditions, its grasping stability still requires further improvement in high-load tasks. Future studies may focus on optimizing the size, shape, or material of the suction cups to increase their adhesive force. Moreover, simplifying the manufacturing process could improve overall accuracy, reduce production time, and decrease labor requirements. Future research will focus on integrating embedded sensors to enable real-time measurement of the soft robotic arm’s motion and deformation, thereby improving its motion precision and control capabilities. We plan to employ CFD simulations and other fluid dynamics techniques in future studies to analyze the effects of water flow on the soft robotic arm’s motion, further refining its design and improving its performance in underwater environments.

In conclusion, the soft arm with a suction cup proposed in this study demonstrates excellent performance in underwater tasks, particularly in terms of flexibility and efficiency in complex environments. Future research could further optimize the suction cup design to enhance load capacity and explore additional application scenarios, such as underwater archaeology, search and rescue, and other tasks in unpredictable environments.

## Figures and Tables

**Table 1:** D-H Parameters Table for the Soft Arm.

i	θ	$d_{j}$	$a_{j}$	$\alpha_{i}$
1	$\phi_{i}$	0	0	−π/2
2	$\theta_{i}/2$	0	0	π/2
3	0	$2r_{i}\sin(\theta_{i}/2)$	0	−π/2
4	$\theta_{i}/2$	0	0	π/2
5	$-\phi_{i}$	0	0	0

## Data Availability

The original data and code supporting the conclusions of this article will be provided by the authors upon request.
